# Rheological analysis of sputum from patients with chronic bronchial diseases

**DOI:** 10.1038/s41598-020-72672-6

**Published:** 2020-09-24

**Authors:** Jérémy Patarin, Étienne Ghiringhelli, Guillaume Darsy, Martinien Obamba, Philippe Bochu, Boubou Camara, Sébastien Quétant, Jean-Luc Cracowski, Claire Cracowski, Matthieu Robert de Saint Vincent

**Affiliations:** 1 Rheonova, domaine universitaire, 1270 rue de la piscine, 38400 Saint Martin d’Hères, France; 2 Service Hospitalo-Universitaire de Pneumologie et Physiologie, Pôle Thorax et Vaisseaux, Centre hospitalier universitaire de Grenoble-Alpes, Boulevard de la Chantourne, 38700 La Tronche, France; 3 Inserm CIC1406, Centre hospitalier universitaire de Grenoble-Alpes, Boulevard de la Chantourne, 38700 La Tronche, France

**Keywords:** Respiratory tract diseases, Biomarkers, Gels and hydrogels, Rheology, Permeation and transport, Biophysical methods

## Abstract

Bronchial diseases are characterised by the weak efficiency of mucus transport through the lower airways, leading in some cases to the muco-obstruction of bronchi. It has been hypothesised that this loss of clearance results from alterations in the mucus rheology, which are reflected in sputum samples collected from patients, making sputum rheology a possible biophysical marker of these diseases and their evolution. However, previous rheological studies have focused on quasi-static viscoelastic (linear storage and loss moduli) properties only, which are not representative of the mucus mobilisation within the respiratory tract. In this paper, we extend this approach further, by analysing both quasi-static and some dynamic (flow point) properties, to assess their usability and relative performance in characterising several chronic bronchial diseases (asthma, chronic obstructive pulmonary disease, and cystic fibrosis) and distinguishing them from healthy subjects. We demonstrate that pathologies influence substantially the linear and flow properties. Linear moduli are weakly condition-specific and even though the corresponding ranges overlap, distinct levels can be identified. This directly relates to the specific mucus structure in each case. In contrast, the flow point is found to strongly increase in muco-obstructive diseases, which may reflect the complete failure of mucociliary clearance causing episodic obstructions. These results suggest that the analysis of quasi-static and dynamic regimes in sputum rheology is in fact useful as these regimes provide complementary markers of chronic bronchial diseases.

## Introduction

Chronic bronchial diseases, viz. asthma, chronic obstructive pulmonary disease (COPD), bronchiectasis, cystic fibrosis (CF) to cite a few, represent a major and growing public health issue^1^. In the lower airways, the inner surface of bronchi is covered by mucus, a hydrated ($\simeq 98\%$ water) cross-linked network of high molecular weight mucins which acts as a physical barrier to inhaled contaminants and is continuously secreted and digested^2^. Although very distinct in origin and mechanisms, chronic bronchial diseases share as common feature a failure of mucus transport, which may lead to mucus overproduction and/or accumulation. In greater or lesser amount, according to their condition, patients produce sputum, a mucus solution with biological material (leucocytes, bacteria, proteins, DNA) therein, and/or partly dehydrated. However, these mucus releases are not always sufficient to contain the accumulation. In the so-called muco-obstructive diseases^3^ (COPD, bronchiectasis, CF, primary ciliary dyskinesia) inflammations and/or infections episodically cause acute crises, called exacerbations.

Since these respiratory diseases are caused by altered mucus transport, a tempting hypothesis is that mucus rheological properties are altered; this is indeed etymologically apparent from the synonym of cystic fibrosis, ‘mucoviscidosis’, which stands for ‘viscous mucosities’ in reference to the very thick appearance of sputa. This assumption motivated pioneer investigations on mucus viscoelasticity starting from the late 1960s^4–7^. The intuited link between sputum rheology and the symptomatic difficulty to clear airways was empirically confirmed^8–12^, and supported the development of therapies relying on mucus fluidisation^13–17^ or mechanical stimulation (physiotherapy, exercise)^18,19^ to improve mucus drainage.

More recently, the potential of sputum rheology as a direct biomarker in CF was brought forward, especially by linking the abnormal viscoelastic properties of CF sputum to secondary infection and inflammation^20^. Macroscopically, the viscoelastic anomaly is attributed to purulence, as mucoid (i.e., not infected or inflamed) CF sputum is essentially indistinguishable from healthy sputum, even though the microstructure is altered^21,22^. Tomaiuolo et al. correlated the bacterial charge with viscoelastic properties in sputa collected from CF patients and showed that the dominant bacterial species involved lead to specific rheological responses^23^. Very recently, a longitudinal study specifically evidenced the rheological signature of exacerbations^24^, showing a strong (and reversible) increase of the storage and loss moduli during the crisis. The exacerbation state also produces a measurable signature in spirometry, through a transient decrease in FEV$_1$, as well as in sputum composition, through a transient increase in solid fraction, but these changes were not considered statistically significant. These correlations support the idea that sputum viscoelasticity could directly characterise the severity and evolution of CF, which raises the practical question of the stability of this biomarker in time. A very recent study actually concluded to the poor statistical reliability of repeated measurements on a group of patients, based on low values of the intraclass correlation coefficients^25^. However, the measurement variability was not compared to the rheological modifications characterising a clinical event. It is thus difficult to conclude on whether the poor correlation reported actually represents a practical limit to sputum rheology in biomedical studies.

Overall, the potential of rheological biomarkers in CF is increasingly acknowledged in the biomedical research community, and it is tempting to generalise this approach to other chronic bronchial diseases if the hypothesis of severity-dependent mucus properties remains true. A first step in this direction was undertaken by Serisier et al., who compared the viscoelasticity levels of CF, COPD, and normal sputa (healthy subjects)^20^. In CF, mucoid sputa have viscoelastic moduli statistically comparable to those of healthy subjects. In contrast, COPD patients feature significantly higher viscoelastic levels even in the absence of inflammation. This result suggests that the linear viscoelastic moduli may not represent characteristic markers of the pathologies and should be complemented with a refined rheological analysis. Indeed, although the stresses and strains involved in bronchial obstruction are not quantified, and possibly not even common to all obstructive diseases, clearance in obstructed states involves strong mechanical solicitations to the mucus when patients expectorate. Cough^26^, for instance, is a turbulent air drag with air flows as high as 100 m s$^{-1}$. Mucus mobilisation in this regime involves rupturing mechanisms, which depend on properties such as adhesion and cohesion^27^, and thus clearly overcome quasi-static shear characterised by the storage and loss moduli. It is therefore tempting to explore the dynamic rheological behaviour of sputum over a wider range of solicitations, towards plastic or even flow regimes, as a first step to assess the relevance and feasibility of large-deformation rheological measurements.

The objective of the present paper is to assess the usability and relative performance, in the general context of chronic bronchial diseases (muco-obstructive or not), of easy-to-access rheological quantities representative of the rich rheological behaviour of sputum. To this aim, we characterise sputum samples produced by healthy volunteers, patients with asthma, COPD, and CF, through quick analyses of fresh samples performed in a clinical environment. We first define rheological observables characteristic of the small- (linear viscoelastic region, LVR) and large-deformation (flow point) regimes. We then evaluate quantitatively the stability (evolution in time) of these rheological parameters, and confirm the existence of a baseline, patient-dependent level. We also characterise the rheological influence of induction, a standard procedure to help sputum production^28^. Comparing these rheological quantities for the four considered populations, we finally demonstrate that they follow segmented viscoelastic levels, and that the flow point very robustly distinguishes the muco-obstructive conditions.

## Results

### Strain sweeps straightforwardly characterise sputum rheology

**Figure 1:**
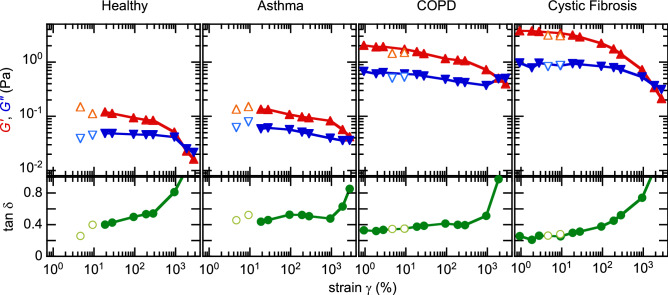
Sputum rheological response to strain. Evolution of the storage ($G', \blacktriangle$) and loss ($G'', \blacktriangledown$) moduli, and the damping ratio ($\tan \delta = G'/G'', \bullet$) with the applied strain, obtained during a strain sweep in oscillatory mode (frequency 0.6 Hz). Open symbols: values obtained in frequency sweeps at 5% and 10% strain, respectively.

The structural and dynamic properties of a sample are solicited in the quasi-static and flow regimes, respectively. To assess both successively on sputum samples, we impose a strain sweep in increasing strain ramps from 0.9% up to 3000%. Figure 1 presents, for each of the four conditions considered, a representative example of the evolution of the storage ($G'$) and loss ($G''$) moduli with strain. The rheological behaviour, reminiscent of soft visco-elasto-plastic solids (gels), is qualitatively similar irrespective of the condition tested. At low strain (generally when $\gamma \leqslant 10\%$), $G'$ and $G''$ are constant and thus represent the quasi-static properties of the material, when the structure remains essentially intact under strain (LVR). In sputa, the mechanical response is dominated by elasticity, $G' > G''$, with a damping ratio, $\tan \delta = G''/G'$, typically within 0.25–0.45. The frequency dependence of the moduli follows a weak power law with comparable exponents for $G'$ and $G''$ (see Supplementary Material S1, Fig. S1), as recently observed in COPD sputum^29^ as well as cultured mucus samples^30^.

When increasing the strain beyond about 10%, $G'$ and $G''$ both decrease and eventually converge above $\sim 1000$% strain. In this regime, the network structure begins to irreversibly disentangle (plastic regime). The extent, amplitude and shape of this regime vary from sample to sample, irrespective of the patient’s condition. In most cases, $G''$ decreases at a rate similar to $G'$ as the shear increases, but it sometimes remains almost constant over the whole plastic range (see Healthy case in Fig. 1), and/or raises and overshoots $G'$ at the end of this range (see COPD case in Fig. 1). This alternative behaviour reflects the gradual unfolding and alignment of the microstructural elements along the shear direction, leading to increased dissipation. As a consequence of this variability in the plastic regime, no general rule can be observed regarding the evolution of $\tan \delta$, which either remains roughly constant or gradually increases. The crossover point (when the strain $\gamma = \gamma _c$, typically within 500–3000%) defines the onset of a viscosity-dominated regime, i.e., flow. Note that this critical point could not always be reached and/or measured reliably as some samples tend to spread out of the measuring cell at high shear.

Besides the general behaviour illustrated in Fig. 1, a minority of samples feature an unexpectedly viscous response with weak critical strain $\gamma _c$ values, around 100% or below. These fluid-like samples are suspected to be mostly constituted of saliva and thus ruled out—we set a cutoff value $\gamma _c = 500\%$, midway between saliva-dominated and sputum-dominated behaviours, below which a sample is considered polluted. Besides, several samples (especially among asthmatics and COPD patients) seem to feature a crossover while $G'$ and $G''$ both increase very abruptly beyond 1000% strain (Fig. S2). The physical meaning of this apparent abrupt transition is not identified and it likely relates to an instrumental artefact. To avoid the risk of undue comparisons, samples featuring this unusual behaviour are set apart from the analysis and discussed separately in Supplementary Material S2.

To sum up, two characteristic strain responses of sputum samples can be extracted unambiguously in the LVR and at the onset of flow (crossover point). We extract the value of $G'$ and $G''$ at $\gamma = 5\%$ (see Methods), which generally falls within (or close to) this first regime and can be measured with reasonable accuracy. Since the linear response is essentially elastic, we focus on $G'$; $\tan \delta$ is also systematically reported as a structural marker of the storage versus dissipation balance. At the crossover, the value of the modulus, $G' = G'' = G'_c$ allows us to calculate the critical stress, $\sigma _c = \sqrt{2} G'_c \gamma _c$, which physically corresponds to the stress needed to make the material flow. We now analyse the robustness of these rheological quantities to characterise a patient’s condition.

### Sputum rheology is temporally stable

The perspective of using sputum rheology in biomedical or clinical research relies on the assumption that the values of the measured rheological quantities are patient-specific, and therefore stable over relatively short times, as long as the patient remains stable.

**Figure 2:**
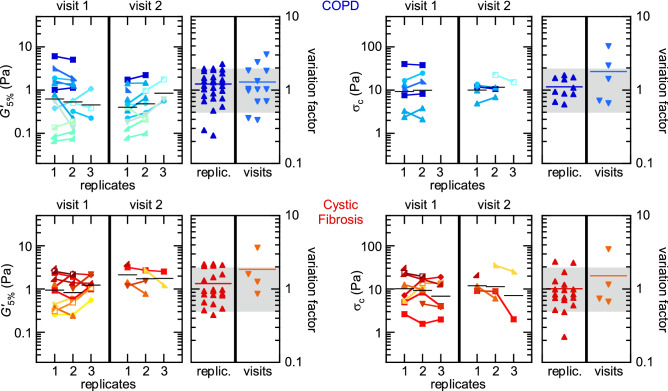
Temporal stability of sputum rheology. Evolution of the linear storage modulus, $G'_{5\%}$, and critical stress, $\sigma _c$, between consecutive replicates and between consecutive visits, for individual COPD (top) and CF patients (bottom). Each connected group of symbols corresponds to a single sputum sample; symbols are associated to individual patients. The horizontal bar in each replicate is the geometric mean. The variation factors are the ratios of replicates $n+1$ and *n* taken individually, and of replicated values in each visit (bar: mean variation factor). The shaded area delimits variations (increase or reduction) by a factor smaller than two.

Figure 2 shows the individual evolution of $G'_{5\%}$ and $\sigma _c$ measured on consecutive measurements, performed on aliquots from the same sample (replicates) on the one hand, and on consecutive samples produced two days apart by the same stable patient on the other hand. The symbols and colours identify individual COPD (top) and CF patients (bottom). In both cases, individual values spread within up to two orders of magnitude in both the linear and crossover regimes. Considering samples individually, Fig. 2 shows that, with few exceptions, variations between consecutive replicates remain tight within a factor of two (i.e., a variation factor comprised between 0.5 and 2, as delimited by the shaded area). The two-days variations are calculated by comparing, for each patient, the average of replicates in visits 1 and 2. Although few patients feature an increase or decrease by a factor of up to 4–5 in isolated cases, a large majority of them are actually stable (within the shaded area), so that the typical relative variations between replicates (same sample) and between visits (same patient, distinct samples) are comparable in amplitude.

The amplitude of the observed variations is, in all cases, very small in comparison with the expected signature of major clinical events. In the linear regime, $G'$ was reported to consistently increase by a factor of around 5 in CF patients when experiencing an exacerbation, and symmetrically decrease back to the base level after the event^24^: this modification of sputum rheology is much bigger than the typical variations observed in the present study. Consecutive measurements can thus be considered stable, both at the sample and at the patient levels, when considering the characterisation of major events.

To assess the statistical robustness of the measurement stability, we performed correlation tests between consecutive replicates and visits when available. Table 1 gathers the Spearman correlation coefficient and the corresponding *p*-value obtained in each case (see also Supplementary Material S3, Table S1). Consecutive replicates (first row) are significantly correlated in all cases ($p < 0.05$), which confirms the strong stability of measurements at the sample level. This stability shows that, within the duration of the set of measurements (up to $\sim$ 1 h after expectoration), sputum samples do not evolve substantially, and repeated comparative measurements can be meaningful if enough sample can be collected.

Between visits (second row), a significant correlation is only found for $G'_{5\%}$ in COPD patients. In all other cases, correlations cannot be confirmed due to the very small population size ($N \leqslant 5$) which does not allow us to draw a statistically robust conclusion. However, considering the moderate two-days variations observed in the majority of samples (Fig. 2), and the statistically robust two-days correlation found in a more favourable case ($G'_{5\%}$ in COPD, with $N = 11$), it is sensible to anticipate the global two-days stability of rheological measurements.

**Table 1:** Results of Spearman correlation tests between the (Visit 1, Replicate 1), (Visit 1, Replicate 2) and (Visit 2, Replicate 1) cases: Spearman coefficient $\rho$, *p*-value and sample size *N*.

Visit 1, Replicate 1 versus	COPD, $G'_{5\%}$	COPD, $\sigma _c$	CF, $G'_{5\%}$	CF, $\sigma _c$
**Visit 1, Replicate 2**				
$\rho$	0.918	0.889	0.694	0.675
*p*	< 0.0001****	0.017*	0.008**	0.048*
*N*	12	6	9	7
**Visit 2, Replicate 1**				
$\rho$	0.556	0.090	1.000	1.000
*p*	0.012*	0.683	0.333	0.333
*N*	11	5	3	3

*$$p < 0.05$$; **$$p < 0.01$$; ****$$p < 0.0001$$.

This result seems to contradict the conclusions of Radtke et al., who rather concluded that the stability of rheological measurements cannot be statistically confirmed^25^. However, their study assessed the measurement stability over a longer time ($7 \pm 1$ days, with an intermediate visit), which makes clinical evolution increasingly likely. Noteworthy, the median values of elastic moduli reported are very similar in the first two visits, but substantially smaller in the third, with poor overlap of the distributions. This study thus does not jeopardise our conclusion that sputum can be considered stable within 2 days.

Sputum rheological stability implies that baseline levels can be defined and considered representative of the stable patient with a given condition. Of course, the robustness of rheological characterisation also relies on standardised protocols, especially during sputum production. In light of this, we now evaluate how rheological measurements are affected by a pre-collection induction.

### Inducing sputum modifies its rheology

A possible practical limitation to sputum analyses is the ability to collect sufficient amount of sample. As a matter of fact, spontaneous production of sputum is only possible with over-secreting patients. To help patients expectorate, induction^28^, which consists in nebulising saline solutions with a mouthpiece, is common practice; it is actually necessary in cases when patients cannot expectorate spontaneously. However, as saline solutions are mucus hydrators, they likely affect sputum rheology^17^. We assessed this effect quantitatively on both COPD and CF sputa, as these two populations were able to expectorate spontaneously.

**Figure 3:**
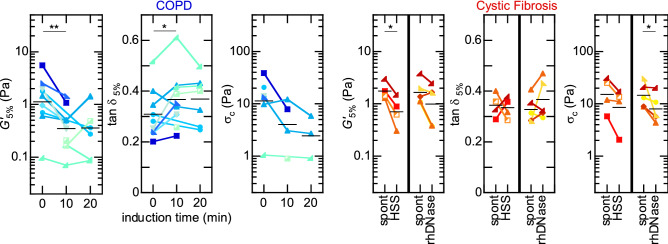
Effect of induction on sputum rheology. Left: Evolution of the linear storage modulus, $G'_{5\%}$, damping ratio, $\tan \delta _{5\%}$, and critical stress, $\sigma _c$, before ($t = 0$, spontaneous expectoration) and after nebulisation of 4.5% HSS, for individual COPD patients. Right: Same rheological parameters, comparing spontaneous expectoration to 4.5% HSS nebulisation (10 min) and to 2.5 mL rhDNase in CF patients. Each connected group of symbols corresponds to consecutive sputum samples collected from the same individual patient; each symbol is a mean over consecutive replicates when available. Horizontal bars: geometric ($G', \sigma _c$) or arithmetic ($\tan \delta$) mean over the corresponding populations. *$p < 0.05$; **$p < 0.01$.

The left panel of Fig. 3 presents the evolution of $G'_{5\%}$, $\tan \delta _{5\%}$ and $\sigma _c$ measured on COPD sputa, collected through several consecutive expectorations before ($t = 0$) and during nebulisation of 4.5% hypertonic saline solution (HSS). Both $G'_{5\%}$ and $\sigma _c$ respectively decrease by a factor of $2.9 \pm 1.8$ ($p = 0.009$) and $3.4 \pm 2.3$ ($p = 0.246$) during the first 10 minutes, and then, on average, saturate. The initial trend is also consistently observed at the individual patient level, except for patients whose sputum already had low rheological base levels. The evolution of $\tan \delta _{5\%}$ is more contrasted, although the average trend shows a slight increase, from 0.31 to 0.37 ($p = 0.043$), which could reflect a mild hydrating effect. Cystic Fibrosis sputa (Fig. 3, right panel) also feature reductions, by factors of $2.6 \pm 1.1$ ($p = 0.014$) and $1.9 \pm 0.7$ ($p = 0.061$), of $G'_{5\%}$ and $\sigma _c$ through HSS induction, and contrasted changes in $\tan \delta _{5\%}$ ($p = 0.836$). HSS nebulisation thus similarly leads to a substantial reduction of the linear and crossover rheological parameters in both pathologies, although more pronounced in COPD than in CF.

For the sake of comparison, recombinant human DNase I (rhDNase, Pulmozyme) was administered to the CF patients during their second visit. RhDNase is a mucolytic drug, widely used in CF treatment, which weakens purulent mucus by cleaving DNA^13,15^. As illustrated in the right panel of Fig. 3, rhDNase affects sputum rheology in a way quantitatively comparable to HSS ($G'_{5\%}$ and $\sigma _c$ are reduced by a factor of $1.9 \pm 1.0$ and $2.2 \pm 1.6$ respectively; $p = 0.053$ and $p = 0.049$).

In summary, HSS induction unambiguously leads to a substantial weakening of the sputum rheological baseline in COPD and CF patients, largely exceeding the measurement variability. Induction should therefore be considered as an influential parameter when comparing rheological measurements in sputa. Consequently, in view of a standardised protocol, even over-secreting patients should undertake induction if their sputum properties are to be compared to those of healthy subjects.

### Sputum rheology characterises chronic bronchial diseases

We now turn back to the comparison of rheological properties for patients with distinct conditions. Considering that a patient can be characterised by the baseline level of his/her sputum rheology, we now wonder if this level is itself determined by the disease. We thus build, for each rheological quantity, the distribution of patients levels in each population.

**Figure 4:**
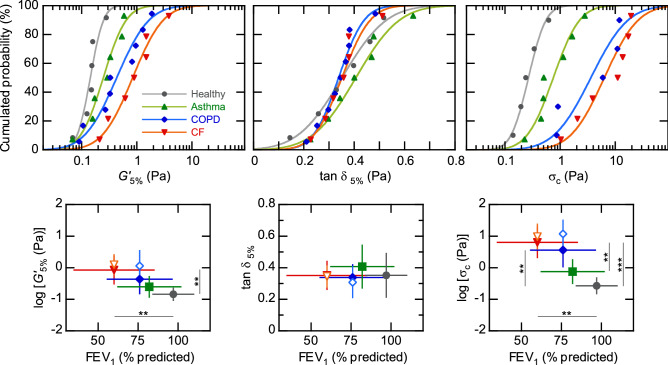
Rheological levels in several bronchial conditions. Top: probability plots of the values of $G'_{5\%}$, $\tan \delta _{5\%}$ and $\sigma _c$ measured from subjects with the four conditions (healthy, $\bullet$; asthma, $\blacktriangle$; COPD, $\blacklozenge$; CF, $\blacktriangledown$). Each symbol corresponds to the per-patient average value after HSS nebulisation; solid line is a fit with Eq. (1). Bottom: expected values and standard deviations ($X_0 \pm \Sigma$) of these rheological parameters, compared by condition, as a function of the corresponding relative FEV$_1$ (mean ± standard deviation). Filled and open symbols depict HSS-induced and spontaneous sputa, respectively. **$p < 0.01$; ***$p < 0.001$.

Given the stability of rheological measurements, each patient’s level is defined by averaging all values obtained after HSS nebulisation. This gives us, for each rheological quantity, a single value per patient and avoids overweighting the most productive ones. The upper panel of Fig. 4 presents these data in the form of probability plots of $G'_{5\%}$, $\tan \delta _{5\%}$ and $\sigma _c$ for the four populations of healthy subjects, asthmatics, COPD, and CF patients. The cumulated probability over all measured quantities is very well adjusted with a cumulative distribution function *E*(*X*) of the form

1 $$\begin{aligned} E(X) = \frac{N}{2}\left( 1+\mathrm {erf}\left( \frac{X-X_0}{\sqrt{2}\Sigma }\right) \right) , \end{aligned}$$

with $\mathrm {erf} (x) = \frac{1}{\sqrt{\pi }} \int _{-x}^x \mathrm {e}^{-t^2} \mathrm {d}t$ the error function, and $X = \log G'_{5\%}$, $\tan \delta _{5\%}$ and $\log \sigma _c$ respectively; $X_0$ and $\Sigma$ are respectively the expected value and the standard deviation. With these variables, the functional form of *E*(*X*) implies that the distributions of $\tan \delta _{5\%}$ on the one hand, and $G'_{5\%}$ and $\sigma _c$ on the other hand, are normal and log-normal, respectively. The calculated expected values ($X_0$) and standard deviations ($\Sigma$) are depicted in the lower panel of Fig. 4; Table 2 summarises the corresponding physical quantities for the whole set of measured rheological parameters.

Figure 4 confirms that sputum rheology can distinguish pathologies. In the linear regime, the elastic moduli decrease as the mean relative FEV$_1$ values increase, and nicely succeed each other from healthy (expected value, $G' = 0.14~\mathrm {Pa}$), asthmatics (0.25 Pa), COPD (0.43 Pa) up to CF patients (0.84 Pa), illustrating a gradually stiffer mucus. However, some overlap exists and only CF and healthy sputa are statistically distinct ($p < 0.01$). The damping ratio $\tan \delta$ remains consistently around 0.35. Thus, even if the gel-type structure of mucus is likely distinct in each condition, which is reflected macroscopically through the distinct $G'$ values, the influence of dissipation is essentially identical. No condition distinguishes by an intrinsically ‘more fluid’ or ‘more solid’ mucus.

At the crossover, the four conditions follow the same ranking sequence than observed on linear moduli, with a smaller overlap between populations. Especially, the $\sigma _c$ values corresponding to COPD and CF patients are distinctly higher (by a factor of 10–20) than those measured in healthy subjects (3.61 and 6.34 Pa compared to 0.27 Pa; $p < 0.01$ and $p < 0.001$ respectively). Asthmatics lie in an intermediate level (0.75 Pa), but closer to the healthy subjects, and statistically distinct from CF patients ($p < 0.01$). The critical stress thus strikingly distinguishes obstructive (COPD, CF) from non-obstructive conditions (asthma, healthy), which highlights the role of mucus dynamic (flow) properties in bronchial obstruction. Table 3 summarises the results of Bonferroni’s all pair comparison tests performed from the ANOVA, and also includes the statistical analysis of FEV$_1$ measurements. These results confirm the enhanced specificity of crossover measurements ($G'_c$ and $\sigma _c$) compared to linear moduli ($G'$ and $G''$ at 5%) and to the FEV$_1$. Sputum rheology can thus much better distinguish the pathological conditions, especially the obstructive ones, than spirometry.

For the sake of comparison, the expected values and standard deviations for spontaneous sputa are also represented in the lower panel of Fig. 4 (open symbols; COPD and CF patients only) and summarised in Table 2. COPD and CF levels become strikingly similar in the linear and crossover regimes.

**Table 2:** Expected and extreme values obtained from the distributions for each rheological quantity measured.

	$G'_{5\%}$ (Pa)	$G''_{5\%}$ (Pa)	$\tan \delta _{5\%}$	$G'_c$ (Pa)	$\sigma _c$ (Pa)
**Healthy, HSS**					
Expected	0.14	0.047	0.35	0.012	0.27
Min–max	0.07–0.28	0.010–0.129	0.14–0.52	0.005–0.036	0.14–0.62
**Asthma, HSS**					
Expected	0.25	0.098	0.41	0.019	0.75
Min–max	0.07–0.59	0.043–0.208	0.22–0.63	0.007–0.068	0.23–2.81
**COPD, HSS**					
Expected	0.43	0.14	0.34	0.097	3.61
Min–max	0.09–1.88	0.03–0.44	0.21–0.48	0.024–0.327	0.87–11.91
**COPD, spontaneous**					
Expected	1.15	0.33	0.31	0.31	11.77
Min–max	0.10–5.54	0.05–1.12	0.20–0.52	0.03–1.08	1.05–38.90
**CF, HSS**					
Expected	0.84	0.28	0.35	0.21	6.34
Min–max	0.21–3.83	0.09–0.76	0.23–0.51	0.02–0.59	1.03–19.33
**CF, spontaneous**					
Expected	1.24	0.41	0.34	0.32	9.68
Min–max	0.23–3.41	0.12–1.17	0.28–0.53	0.04–0.76	1.43–26.00

**Table 3:** Results of the ANOVA: *p*-values obtained from Bonferroni’s all pair comparison test.

	Asthma	COPD	Cystic fibrosis
**Healthy**			
FEV$_1$	1	0.360	0.009**
$G'_{5\%}$	1	0.157	0.009**
$G''_{5\%}$	0.719	0.117	0.004**
$G'_c$	1	0.029*	0.0008***
$\sigma _c$	0.579	0.004**	0.0002***
**Asthma**			
FEV$_1$		1	0.132
$G'_{5\%}$		1	0.096
$G''_{5\%}$		1	0.156
$G'_c$		0.157	0.009**
$\sigma _c$		0.095	0.005**
**COPD**			
FEV$_1$			0.545
$G'_{5\%}$			0.896
$G''_{5\%}$			0.636
$G'_c$			1
$\sigma _c$			1

*$$p < 0.05$$; **$$p < 0.01$$; ***$$p < 0.001$$.

## Discussion

To summarise, we analysed the quasi-static and dynamic rheological properties of sputum in a set of chronic bronchial diseases. In these samples, the presence of viscoelastic inclusions of size similar to the rheometer gap prevents meaningful rheological measurements, making homogenisation a prerequisite. Although the effects of this process on sputum rheology are yet to be investigated more thoroughly, our tests suggest that vortex homogenisation rather preserves the average gel properties. However, the pre-shearing of sputum samples can induce some strain-hardening (Fig. S3), which prevents the direct comparison between homogenised and unprocessed samples. It is thus essential to systematically process the samples according to the same protocol.

We identified two physical quantities characterising the quasi-static and dynamic rheological properties, $G'_{5\%}$ and $\sigma _c$, which can be obtained through a standard strain sweep procedure. The stability of these characteristics is proven robust at least over replicated measurements, and can be reasonably expected in consecutive samples provided that the patient remains stable. We also demonstrated that HSS induction substantially alters the sputum rheological properties and should therefore be accounted for when comparing patients.

Taking this effect into consideration, we thus compared the rheological data obtained from healthy subjects or with either muco-obstructive (COPD, CF) or non-obstructive (asthma) chronic bronchial diseases. We found a weak segmentation of the populations according to their sputum linear moduli, showing a gradual increase from healthy subjects, asthmatics, then COPD and finally CF patients, the extreme conditions being typically one order of magnitude apart. This segmentation concerns both the storage and loss moduli alike, so that the damping ratio remains fairly identical in all populations. Linear rheology thus proves sensitive to the specific structure of each mucus, although structural differences between them may as well be exceeded by external elements, such as the unusual presence of biological material, that were not considered here.

Interestingly, the crossover distinguishes frankly the obstructive patients from the non-obstructive and healthy cases, with critical stress values typically around 5–10 Pa and below 1 Pa, respectively. On the bronchial epithelium, the force a single cilium can exert is estimated to 1 pN^31^, corresponding to a stress in the order of 1 Pa for the cilium to penetrate into the mucus layer by $\simeq 1 \mu$m. To set mucus in motion at large scale, cilia must first grab the mucus layer, and thus deform it plastically. The stress they exert should therefore locally exceed the yield stress of mucus, which can be identified to $\sigma _c$. The comparison of this estimate with our measurements suggests that, in chronic respiratory diseases when the mucus critical stress is increased, the effectiveness of ciliary transport falls down as cilia fail to grab the mucus layer. Especially, when the critical stress exceeds the stress cilia can exert, the mucociliary clearance would totally stop, thus favouring the bronchial obstruction by accumulated mucus. Although this order-of-magnitude estimate should be confirmed by refined investigations on the mucus–cilium interaction^31–34^, it nonetheless highlights the relevance of sputum dynamic properties, in complement with quasi-static properties, as possible markers of chronic bronchial diseases.

## Methods

### Clinical protocol

We conducted a prospective, interventional, single-group assignment study. The study protocol was approved by the local ethic committee (Comité de protection des personnes Sud Est V; Grenoble, France IRBN6705) and registered to the national authority (ANSM) under RCB 2014-A01858-39, and research was performed in accordance with the relevant guidelines and regulations. All subjects were informed about the nature and purpose of the study. Each subject provided written informed consent before enrolment. Forty-five subjects (11 healthy volunteers, 12 asthmatics, 11 COPD and 11 CF patients) were invited at the Centre d’Investigation Clinique in Grenoble University Hospital on two occasions. During the first visit ($t_0$), patients able to expectorate spontaneously (COPD and CF) were asked to, then all participants had one or more induced expectoration after 5, 10, 15, and/or 20 min nebulisation of a 4.5% hypertonic saline solution (HSS). The same procedure was repeated during the second visit ($t_0 + 48$ h), except for CF patients who were asked to expectorate without HSS nebulisation, 1 hour after inhaling 2.5 mL rhDNase. All patients were $\geqslant 18$ in age and in stable state (no acute exacerbation during the preceding month), with relative FEV$_1 > 50\%$ (asthmatics) or $> 40\%$ (COPD, CF); see Supplementary Material S4, Table S2, for information on patients demographics and pulmonary function. Detailed protocol and eligibility criteria are provided in clinicaltrials.gov under NCT02682290^35^.

### Sample preparation

Collected samples are highly heterogeneous as they consist of thick mucous inclusions within a viscoelastic matrix; in addition, saliva, and bubbles inevitably collected with the sputum samples form a watery layer on top of them. Immediately after expectoration, the saliva is removed by gently aspirating the lower (sputum) phase with a positive displacement pipette. Sputum is transferred in a flat-bottom flask and homogenised in vortex, typically below 6000 rpm to ensure shear rates remain under 50 s$^{-1}$. This step allows to fractionate mucous inclusions without damaging the viscoelastic properties of sputum (Supplementary Material S5, Fig. S3)^36^.

After homogenisation, sputum samples are divided into aliquots (at least two, whenever possible) in order to perform replicate measurements in a row.

### Rheological measurements

Rheological measurements were performed immediately on a dedicated device designed and built by Rheonova, Rheomuco (Fig. S4), which consists of a strain-controlled, rotational rheometer, working in oscillatory mode and equipped with rough plane geometries (see Supplementary Material S6 for details).

Immediately after preparation, samples were transferred onto the rheometer’s geometry with a positive displacement pipette. Sample positioning was checked for accurate centring and excess sample was removed when applicable. The temperature within the gap is regulated at $37 \pm 1\,^{\circ }$C.

A full set of measurements consists of two consecutive frequency sweeps (0.1–5 Hz) at 5% and 10% strain, followed by a strain sweep (0.9–3000%) at 0.6 Hz. Each set of measurements typically takes 20 minutes per replicate (including sample positioning and withdrawal), and produces the storage and loss moduli, $G'$ and $G''$, as a function of frequency and strain respectively. Moduli in the linear regime were directly extracted from the frequency sweep at 5% as they correspond to fresh samples. The crossover point was estimated by interpolation of the strain sweep curves.

Finally, the consistency of the values obtained at 5% strain from the frequency and strain sweeps was systematically controlled, measurements differing by a factor of two or above were not considered (represent $\sim 10 \%$ of total). Two samples featuring unexpectedly high linear moduli (one order of magnitude higher than other samples from the same patient) and a linear response up to 200% strain were also ignored.

### Statistical analyses

The following analyses were performed:

- *Rheological stability*—Spearman’s rank correlation tests on raw values, comparing on the one hand replicates 1 and 2 in visit 1, and on the other hand the first replicate in visits 1 and 2. This test does not require the normality of the distribution and was thus performed with raw values. Intraclass Correlation Coefficients (ICCs) were also calculated on both raw and log-transformed values (Supplementary Material S3);
- *Effect of induction*—Paired Student’s *t*-test between spontaneous and induced sputa (bilateral after HSS, unilateral after rhDNase as rhDNase has a documented reducing effect on sputum rheology^13–15^), on values averaged (over replicates) then log-transformed ($G'_{5\%}$ and $\sigma _c$ only);
- *Characterisation of diseases*—One-way Analysis of variance (ANOVA) with Bonferroni’s all pair comparison test after (i) per-patient all-measurements averaging, and (ii) log-transformation for log-normally-distributed quantities (all but $\tan \delta$).

## Supplementary information

Supplementary Information.

## Data Availability

The datasets generated and analysed during the present study are available from the corresponding author on reasonable request.
