# Supplementary material for: Rheological analysis of sputum from patients with chronic bronchial diseases
Source: Sci Rep. 2020 Sep 24;10:15685. doi: 10.1038/s41598-020-72672-6 (PMC7518272; doi:10.1038/s41598-020-72672-6)
Supplement: Supplementary file 1 — Supplementary Information. [file 41598_2020_72672_MOESM1_ESM.pdf]

Rheological Analysis of Sputum from Patients with Chronic Bronchial Diseases
Supplementary Material

Jérémy Patarin, Étienne Ghiringhelli, Guillaume Darsy, Martinien Obamba, Philippe Bochu, Boubou Camara, Sébastien Quétant, Jean-Luc Cracowski, Claire Cracowski, and Matthieu Robert de Saint Vincent

S1 Frequency sweeps

In oscillating rheology, frequency sweep is a common way to determine the viscoelastic moduli in the LVR. In soft viscoelastic gels, G' and G'' feature a frequency dependence in the form of a weak power law, $G', G'' \sim f^{\alpha}$, with α comprised between 0 (Hookean solid, $G' \sim f^0$ and $G'' = 0$) and 1 (Newtonian liquid, $G' = 0$ and $G'' \sim f^1$). The exponent α is related to the dissipation ratio through $\tan\delta = \tan\left(\frac{\alpha\pi}{2}\right)$ [1].

Figure S1 shows the frequency dependence of G' and G'' of representative sputum samples (the same as in Fig. 1). Over the investigated range, they follow parallel power-law behaviours, as illustrated by the constant value of $\tan\delta$, and are very well adjusted by power-law fits with $\alpha = \frac{2}{\pi}\arctan(\tan\delta)$.

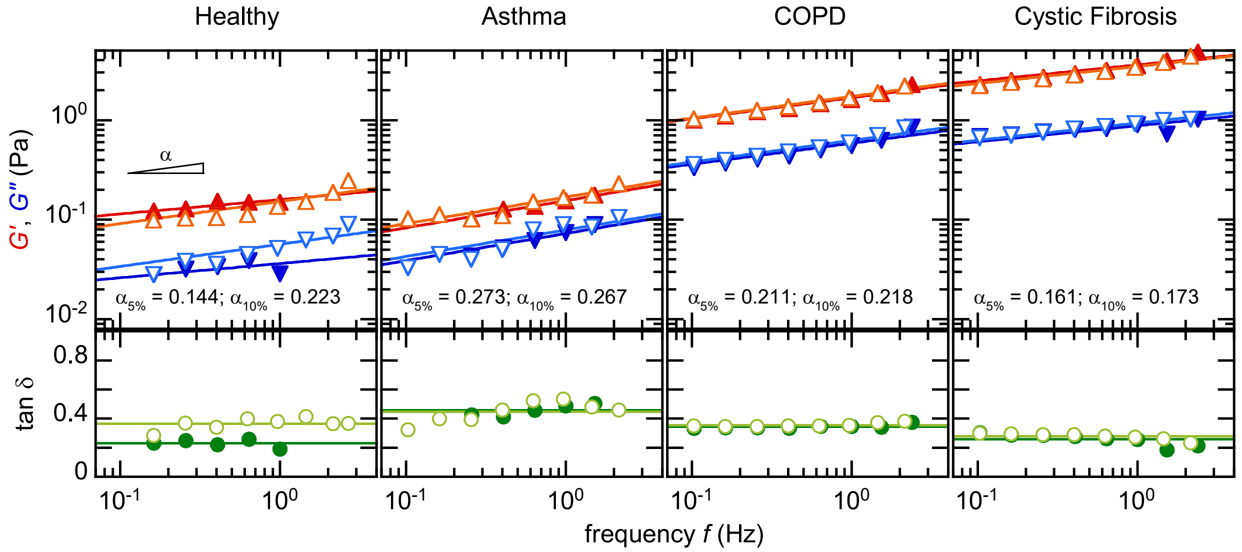

Figure S1: Frequency sweeps in sputum samples. Evolution of the storage (G', ▲) and loss (G'', ▼) moduli, and the damping ratio ($\tan\delta = G'/G''$, ●) with the frequency, obtained during two consecutive frequency sweeps at 5% (filled symbols) and 10% strain (open symbols). Solid lines are power-law fits, $G', G'' \sim f^{\alpha}$ with α fixed from the mean value of $\tan\delta$.

S2 Rising crossovers

Some sputum samples feature unusual behaviour at large strain, with an abrupt rise of both G' and G'' beyond 1,000%, and an apparent crossover within this rising trend, which we refer to as 'rising crossover' (Fig. S2 (a)). The occurrence of this behaviour varies among conditions, from 3% of total large deformation measurements in CF patients to 50% in asthmatics and COPD patients.

From a rheological standpoint, such rise would correspond to a strain-induced structuring of the material, which is rather untypical for cross-linked gels although some stress-hardening behaviour

has been reported in mucin gels [2]. As a matter of fact, we noticed that some samples featuring this behaviour tend to mildly roll themselves up under these large strains, and thus lose their initial pancake-like shape. This rolling effect might result in increased stresses, which then translate into increased moduli.

Although not likely related to the sputum structure itself, this effect is nevertheless a macroscopic signature of the large-deformation behaviour of the corresponding sputum samples, and one may wonder if taking these rising crossovers into consideration (either by mistake or intentionally) drastically changes the calculated critical stress. Indeed, the increasing moduli will mechanically lead to higher G'_c, and therefore higher σ_c values, yet does this increase substantially affect the distributions found without including these data?

The top graph of Fig. S2 (b) presents the probability plots of σ_c for the 4 conditions, including the rising crossovers, and adjusted with an error function (Eq. 1, see article). The adjusted distributions without the rising crossover (same as in Fig. 4 in the article) are also plotted in dashed lines for comparison. Setting aside the CF patients which are unchanged (no rising crossover was observed in induced CF sputa), taking into account the rising crossovers slightly shifts the distributions towards higher σ_c values. All expected values increase by a factor of 1.4–1.5, which preserves the rank observed between the 4 conditions, $\sigma_c^{\text{healthy}} < \sigma_c^{\text{asthmatic}} < \sigma_c^{\text{COPD}} \simeq \sigma_c^{\text{CF}}$ (Fig. S2 (b), bottom).

More subtly, the distributions including the rising crossover tend to favour σ_c values in the range of 0.5–5 Pa, which leads to enlarged distributions towards the higher end in the healthy and asthmatic cases, and conversely, narrowed distribution towards the lower end for COPD patients. This relatively universal range advocates in favour of the hypothesis of sample folding, as we would expect this change in sample geometry to relate with curvature, and thus interfacial effects, rather than sample microstructure.

Nevertheless, modifications in the distributions remain relatively small compared to the gap between populations (Fig. S2 (b), bottom). Including these rising crossovers in the analysis would therefore not substantially affect the conclusions of the present study.

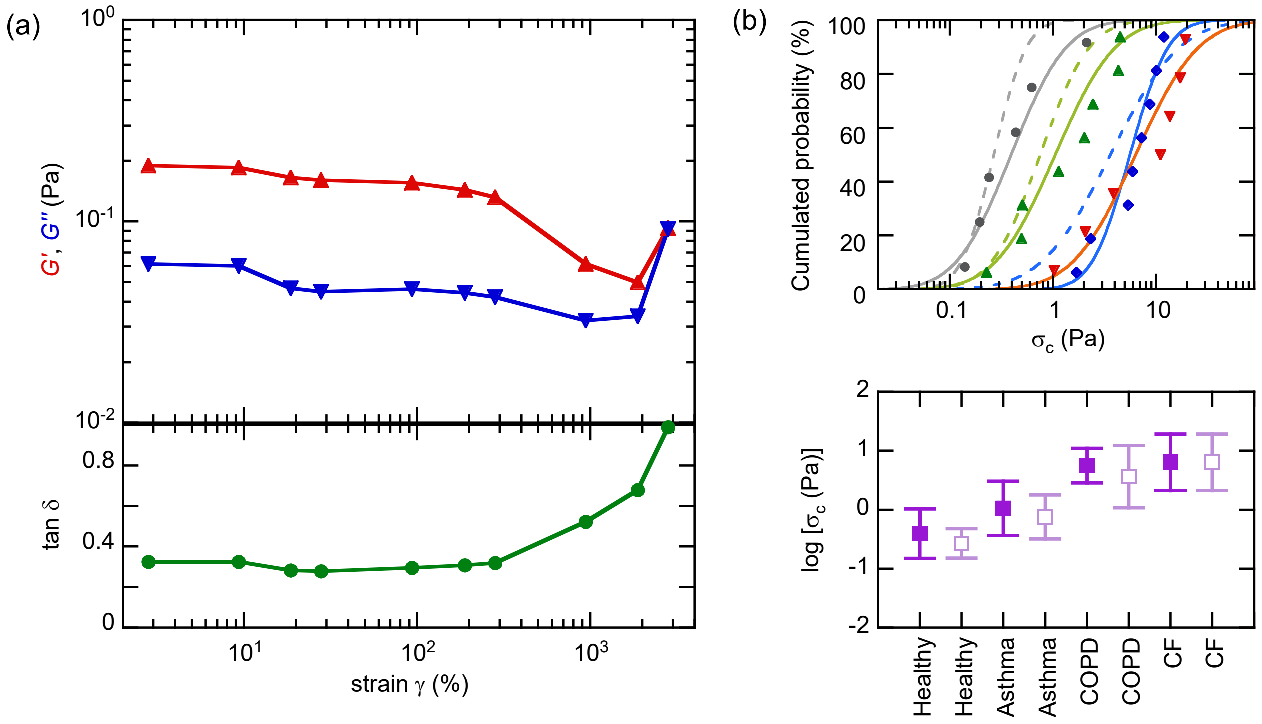

Figure S2: Rising crossover. (a) Example of strain sweep curve featuring a rising crossover (G', ▲; G'', ▼; $\tan\delta = G'/G''$, •). The sputum sample was collected from a COPD patient after a 10-min induction. (b) Modified distributions of σ_c when including the rising crossover data. Top: Probability plots for the four conditions; individual patients data are adjusted with Eq. 1 (solid lines), the fits obtained when ignoring rising crossovers (see Fig. 4) are also represented (dashed lines). Bottom: Corresponding expected values and standard deviations when including (filled symbols) and excluding (open symbols; see Fig. 4) the rising crossovers.

S3 Intraclass Correlation

When assessing the stability of measurements, a standard procedure is to calculate the Intraclass Correlation Coefficient (ICC). However, ICC relies on the assumption that measurements are normally distributed. This hypothesis is difficult to ascertain in our stability measurements as (i) some patients are over-represented due to abundant replicates, and (ii) some comparisons are under-sampled ($N \leqslant 5$, especially between visits 1 and 2). This is the reason why we tested Spearman correlation, which does not require normality, instead. Nevertheless, following Radtke *et al.* [3], and considering that within the subject populations $G'_{5\%}$ and σ_c follow log-normal distributions (Fig. 4), we calculated the ICCs based on both raw and log-transformed values.

Table S1 gathers ICCs calculated using a two-way mixed model ICC(3,1) [4]; interpretation is based on Ref. [5]. Overall, the good correlation obtained with the Spearman test is retrieved, which confirms the statistical concordance of successive measurements.

Visit 1, Replicate 1 *vs.*		COPD, $G'_{5\%}$	COPD, σ_c	CF, $G'_{5\%}$	CF, σ_c
Visit 1, Replicate 2	raw	0.955	0.961	0.831	0.700
	log	0.910	0.947	0.892	0.763
	interpretation	excellent	excellent	excellent	good/excellent
	N	12	6	9	7
Visit 2, Replicate 1	raw	0.532	0.344	0.940	0.760
	log	0.744	0.296	0.982	0.752
	interpretation	fair/good	poor	excellent	excellent
	N	11	5	3	3

Table S1: ICCs calculated between the (Visit 1, Replicate 1), (Visit 1, Replicate 2) and (Visit 2, Replicate 1) cases, from raw and log-transformed measurements. Interpretation: ICC < 0.40 poor; 0.40–0.59 fair; 0.60–0.74 good; 0.75–1.00 excellent.

S4 Patients demographics and pulmonary function

A total of 45 subjects were recruited by the Centre d'Investigation Clinique in Grenoble University Hospital. Among the subjects recruited, several were not able to expectorate, and/or the sputum rheological measurement could not be exploited. Table S2 summarises the demographic and pulmonary function data for the population retained in the rheological study. Note that the FEV_1 remained stable between visits 1 and 2 for all patients.

Condition	N recruited	N	(m/f)	age	(min–max)	relative FEV_1 (%)	(min–max)
Healthy	11	6	(1/5)	43 ± 17	(26–68)	97 ± 13	(74–109)
Asthma	12	9	(3/6)	43 ± 12	(28–68)	82 ± 20	(58–111)
COPD	11	10	(6/4)	65 ± 12	(51–90)	76 ± 20	(36–101)
CF	11	11	(9/2)	33 ± 8	(20–42)	60 ± 25	(28–101)

Table S2: Main demographics and pulmonary function characteristics of the considered population. Age and FEV_1 are given as mean $\pm$ standard deviation.

S5 Sputum homogenisation

Visually, sputum samples consist of highly polydisperse inclusions within a gel matrix. The inclusions are thicker than the matrix, and can reach sizes comparable to the gap width, which makes their contribution dominant in the sample rheological response. This is illustrated in the left panel of Fig. S3 (a), where the linear storage and loss moduli of three aliquots from the same CF sputum sample are compared. The very high dispersion of data reflects a variable proportion of inclusions within the gap. In contrast, vortexing the sample allows to split up the inclusions, and makes them

more homogeneously distributed at the gap scale (right panel). The dispersion of measured linear moduli is strongly reduced. In addition, the average values are identical in the unprocessed and vortexed cases, which suggests that, overall, the vortex homogenisation is not destructive for the gel network.

To assess the onset of gel structure damaging, we performed pre-shear tests on two vortexed sputum samples. The samples are submitted to oscillatory shear, successively at 1, 10, and 100 s^{-1}, exerted with an ARES-G2 rheometer (TA Instruments); the viscoelastic moduli are characterised before the test and between each step. As shown in Fig. S3 (b), G' and G'' both increase up to 10 s^{-1}, then drop between 10 and 100 s^{-1}, similarly for the two samples. This decrease in G' and G'' corresponds to a breakup of the gel structure. More systematic measurements allowed us to determine the critical rate to be around 50 s^{-1} for sputum samples. For comparison, the shear rates imposed in our homogenisation protocol are rather close to 15 s^{-1}.

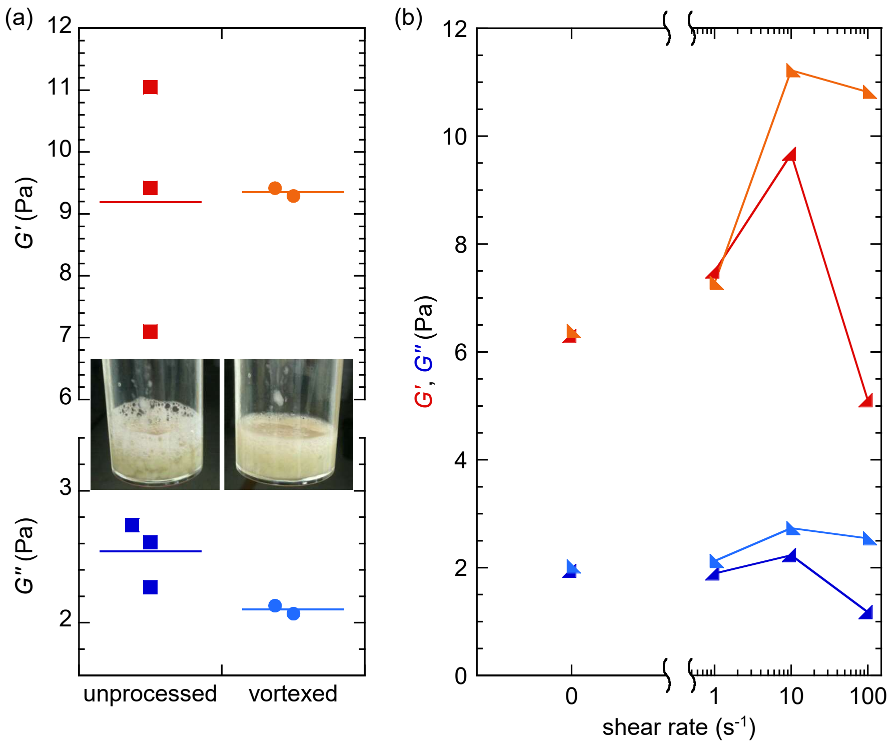

Figure S3: Effect of vortex homogenisation on sputum linear rheology. (a) Comparison of G' and G'' obtained on three unprocessed (left) and two vortexed (right) aliquots. Horizontal bars correspond to the arithmetic mean. (b) Evolution of the viscoelastic moduli of two vortexed sputum samples after pre-shear cycles with increasing shear rates from 0 to 100 s^{-1}. The same symbol refers to a same sample.

S6 Rheomuco

The present work was carried out with a rheometer conceived for the analysis of human secretions, with emphasis on bronchial mucus samples. The analysis of fresh medical material in hospitals raises practical constraints as the specialised laboratory environment (vibration-free table, dust- and oil-free compressed air supply, etc.) usually required for rheometry measurements may not be available. This device, Rheomuco, was developed by Rheonova (Grenoble, France) to overcome these constraints. The present study was performed using a fully operational prototype of this device (Fig. S4).

Rheomuco is a rotational rheometer designed to operate in oscillatory mode, within the 0.1–10 Hz range, and with controlled angular displacement from 10^{-4} to 5 rad. The deformation is imposed by a servomotor, and the torque is measured through a torsion bar with a magnetic bearing to reduce friction without the need for air cushioning. A pair of rough plane geometries machined in Polyoxymethylene with rough surfaces (25 mm diameter; the roughness is made with a square array

of pyramidal structures of height 250 μm and width 500 μm) were used. The gap between the facing geometries is adjustable depending on the sample volume (set at 1 mm for 680 μL).

The strain sweep curve (see Fig. 1) is then analysed automatically. In the LVR, the values of G' and G'' at the viscoelastic plateau, when detected, are extracted together with $G'_{5\%}$ and $G''_{5\%}$, and referred to as G'_p and G''_p, respectively. The crossover point is obtained by interpolation of the G' and G'' curves in double logarithmic scale.

Following current practices in some industrial sectors (adhesives, cosmetics), Rheomuco also calculates two pooled rheological quantities based on these physical values: the so-called "Tack", $\tan \delta_p / G'_p$, empirically relates to the adhesiveness of the sample [6]; and the so-called "Elastic Force", $G'_p \times \sigma_c$, approximates the amount of elastic energy stored by the sample up to the onset of flow [7].

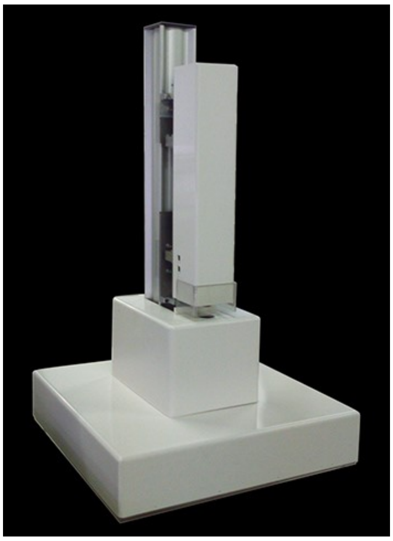

Figure S4: Photograph of the Rheomuco prototype, used in the present study.

References

[1] Nettle, C. *et al.* Linear rheology as a potential monitoring tool for sputum in patients with Chronic Obstructive Pulmonary Disease (COPD). *Biorheology* **54**, 67–80, DOI: 10.3233/BIR-17141 (2018).

[2] Taylor, C., Draget, K. I., Pearson, J. P. & Smidsrød, O. Mucous systems show a novel mechanical response to applied deformation. *Biomacromolecules* **6**, 1524–1530, DOI: 10.1021/bm049225i (2005).

[3] Radtke, T. *et al.* The many ways sputum flows – Dealing with high within-subject variability in cystic fibrosis sputum rheology. *Respir. Physiol. Neurobiol.* **254**, 36–39, DOI: 10.1016/j.resp.2018.04.006 (2018).

[4] Shrout, P. E. & Fleiss, J. L. Intraclass correlations: uses in assessing rater reliability. *Psychol. Bull.* **86**, 420, DOI: 10.1037/0033-2909.86.2.420 (1979).

[5] Cicchetti, D. V. Guidelines, criteria, and rules of thumb for evaluating normed and standardized assessment instruments in psychology. *Psychol. Assess.* **6**, 284, DOI: 10.1037/1040-3590.6.4.284 (1994).

[6] Deplace, F. *et al.* Fine tuning the adhesive properties of a soft nanostructured adhesive with rheological measurements. *J. Adhesion* **85**, 18–54, DOI: 10.1080/00218460902727381 (2009).

[7] Bourdon, F. & Meunier, S. Process for evaluating the mechanical performance of a filler gel. International Patent WO2016150974A1. Available from: https://patents.google.com/patent/WO2016150974A1/en (2016).
